# Dual Graphene Oxide–Based Multigene Delivery for Cancer Elimination via Stromal and Immune Reprogramming

**DOI:** 10.1155/ijbm/3462859

**Published:** 2026-09-06

**Authors:** Francesca Grilli, Sadman Sakib, Fabio Variola, Shan Zou

**Affiliations:** ^1^ Metrology Research Centre, National Research Council of Canada, 100 Sussex Drive, Ottawa Ontario, K1A 0R6, Canada, nrc-cnrc.gc.ca; ^2^ Ottawa-Carleton Institute for Biomedical Engineering (OCIBME), 161 Louis Pasteur, Ottawa Ontario, K1N 6N5, Canada; ^3^ Department of Mechanical Engineering, University of Ottawa, 161 Louis Pasteur, Ottawa Ontario, K1N 6N5, Canada, uottawa.ca; ^4^ Department of Chemistry, Carleton University, 1125 Colonel By Drive, Ottawa Ontario, K1S 5B6, Canada, carleton.ca

## Abstract

Cancer therapies are often limited by poor cellular specificity and tumor microenvironment‐mediated immunosuppression. We developed a dual‐function graphene oxide (GO)–based gene delivery platform combining direct cancer cell elimination with stromal and immune reprogramming. CD47‐targeting siRNA and ANT1‐encoding plasmid DNA selectively induced apoptosis in tumor cells, while TGFβ‐targeting siRNA altered cancer‐associated fibroblasts (CAFs) phenotype and was associated with a shift in macrophage polarization toward a pro‐inflammatory, tumor‐restraining phenotype. We evaluated this approach in a 3D multicellular lung cancer model consisting of A549 cells, human lung fibroblasts, and THP1‐derived macrophages. TGFβ silencing alone modulated stromal and immune features without direct cytotoxicity, whereas CD47/ANT1 modulation reduced overall cell viability to ∼51%. When both therapeutic functions were applied together, overall cell viability was further reduced to ∼29%. EGFR‐targeted GO‐CD47‐ANT1 (GE11‐peptide modified formulation) enhanced tumor‐cell uptake and selective apoptosis in CK7^+^ cancer cells to 91%, while sparing CK7^−^ nonmalignant populations (16%). Mechanistically, combined therapy suppressed immunosuppressive macrophage markers (CD163, IL10, TGFβ), restored epithelial integrity (E‐cadherin 3.5‐fold), and attenuated CAF activation (αSMA 0.3‐fold; vimentin 0.6‐fold). GE11 peptide functionalization shifted nanocarrier internalization toward EGFR‐mediated cancer cell uptake, minimizing off‐target delivery to fibroblasts and macrophages. These findings demonstrate that synergistic modulation of cancer cells and the tumor microenvironment is essential for robust and selective tumor elimination. Our study establishes a modular, mechanism‐informed GO platform integrating dual therapeutic functions—targeted cancer cell killing and microenvironmental reprogramming—highlighting its translational potential in physiologically relevant tumor models.

## 1. Introduction

Despite advances in chemotherapy, radiotherapy, and immunotherapy for the treatment of cancer, many clinical interventions remain limited by systemic toxicity, incomplete tumor specificity, and the emergence of therapy resistance [1, 2]. Gene therapy is gaining attention due to its potential for high tumor specificity and reduced off‐target effects [3–8]. Contemporary gene delivery strategies exploit the precision to eradicate malignant cells while sparing healthy tissues selectively. However, the efficacy and durability of these approaches are inextricably shaped by the stromal and immune context surrounding the cancer, where tumor cells coexist with cancer‐associated fibroblasts (CAFs), endothelial cells, immune cells, extracellular matrix (ECM) components, and a diverse array of soluble mediators [3, 9–12]. Cell–cell and cell–matrix interactions within this ecosystem govern nearly all steps of tumor development, from proliferation and invasion to immune evasion and metastatic dissemination. Cell–cell communication via adhesion molecules and secreted factors between cancer cells and immune cells, such as tumor‐associated macrophages (TAMs), influences tumor growth and metastatic potential. Malignant cells reprogram macrophages via cytokines, leading to the generation of TAMs that support angiogenesis, degrade the ECM via proteases, and suppress cytotoxic T cell activity, facilitating immune evasion and metastasis [13].

CAFs are generated when resident fibroblasts become activated within the tumor milieu, acquiring a contractile and secretory phenotype that fuels tumor growth, invasion, metastasis, and therapy resistance. Dysregulated ECM remodeling by CAFs can alter ECM stiffness and composition, promote metastasis, and inhibit immune cell infiltration. Matrix proteins like tenascin‐C and osteonectin have been shown to induce matrix remodeling that inhibits CD8^+^ T cell infiltration and supports macrophage polarization toward immunosuppressive phenotypes in breast and colorectal cancers [14].

3D co‐cultures of lung cancer spheroids with CAFs demonstrated a significant increase in cancer spheroid growth and a rise in invasive protrusions compared to monocultures, linked to fibroblast–cancer interactions and fibroblast‐mediated ECM remodeling [12, 15, 16]. Moreover, in vitro co‐culture experiments of cancer and immune cells and in vivo tumor models elucidate how tumor‐derived signals regulate immune responses, modulating them to favor cancer proliferation and invasion [9, 17, 18]. Therefore, increasing evidence now highlights the profound impact of these interactions on patient prognosis and therapeutic response [12, 18–21], underscoring the need for therapeutic strategies that not only target cancer cells directly but also account for the microenvironmental constraints imposed by their surrounding cues.

Lung cancer, one of the deadliest malignancies, is heavily infiltrated by myeloid cells (i.e., granulocytes, monocytes, macrophages, and dendritic cells [DCs]) [22, 23], which regulate immune suppression, angiogenesis, metastasis, and therapy resistance. In solid tumors, macrophages often represent the largest myeloid population, comprising up to 50% of the tumor mass in lung cancer [9, 23]. Recruited monocytes can differentiate into macrophages spanning a functional spectrum, ranging from pro‐inflammatory, tumor‐restraining M1 states to immunosuppressive, tumor‐promoting M2 phenotypes. It has been shown that macrophages in the presence of cancer cells closely align with the M2‐like phenotype and strongly promote tumor progression via proliferation, angiogenesis, ECM remodeling, and immunosuppression [22–24]. M2‐like macrophages correlate with poor response to standard therapies and, for this reason, have emerged as a promising therapeutic target [25–28], with controlled tumor progression in murine models and patients, resulting in prolonged survival [27, 29]. These findings support the potential of harnessing macrophage biology as a strategy to enhance cancer elimination.

Among the many regulators of tumor survival, cluster of differentiation 47 (CD47) is a key immune checkpoint that engages SIRPα on macrophages to suppress phagocytosis [30, 31]. Overexpression of CD47 in cancer cells constitutes a fundamental mechanism of immune evasion, and its inhibition restores macrophage recognition and phagocytic clearance of tumor cells. Conversely, adenine nucleotide translocator 1 (ANT1), a mitochondrial protein involved in metabolic regulation, is frequently downregulated in cancer cells. Its re‐expression disrupts mitochondrial function and promotes apoptosis, exploiting cancer‐specific metabolic vulnerabilities [32].

In our previous work [25, 33, 34], we established a graphene oxide (GO) nanocarrier system (∼100 nm lateral size, 1 nm thickness; modified with polyethylene glycol [PEG] and PAMAM dendrimer) capable of efficiently loading and delivering multiple genetic payloads. Due to its large surface area, abundant oxygen‐containing groups, and strong π‐π and electrostatic interactions, GO carriers allow multiple functionalization to protect nucleic acids while achieving high transfection efficiency and cancer‐selective activity [35–38]. Functionalized GO‐PEG‐PAMAM nanocarriers remain colloidally stable at physiological pH (∼7.4), as PEG provides steric stabilization while the PAMAM dendrimer contributes a positively charged corona that limits aggregation and reduces nonspecific protein adsorption. This stability is associated with low to negligible cytotoxicity at concentrations up to 5 μg/mL, supporting safe use under physiological conditions [39–41].

This platform enabled co‐delivery of CD47_siRNA and ANT1_pDNA to selectively reduce cancer cell survival in 3D lung cancer‐stromal‐immune models comprising lung cancer cells, fibroblasts, and monocyte‐derived macrophages. This dual‐targeted approach induced substantial apoptosis in cancer cells (up to 75%) while exhibiting minimal toxicity toward healthy cells (∼35% apoptosis), demonstrating selective elimination driven by coordinated immune modulation together with ANT1‐mediated apoptosis.

Building on this foundation, we now aim to enhance cancer cell elimination further while minimizing collateral effects on stromal and immune populations by incorporating an additional therapeutic target deeply intertwined with cancer regulation. Transforming growth factor‐β (TGFβ) is a multifunctional cytokine produced by tumor cells, immune cells, and CAFs. It drives the activation of fibroblasts into CAFs and stimulates ECM deposition. It also promotes the proliferation and migration of cancer cells while impairing immune cell activity, including that of macrophages [19, 42–44]. Elevated TGFβ levels correlate strongly with poor prognosis, metastatic progression, and resistance to chemo‐ and immunotherapies. Accordingly, therapeutic inhibition of TGFβ signaling has been shown to suppress CAFs activation, normalize the ECM, and sensitize tumors to otherwise ineffective treatments by dismantling pro‐survival and immunosuppressive signals [45–48].

Building on our previously established GO‐based delivery platform [37], we now incorporate an active‐targeting component by functionalizing GO‐PEG‐PAMAM with GE11, a 12‐amino‐acid peptide that selectively binds the epidermal growth factor receptor (EGFR), which is overexpressed in lung adenocarcinoma cells [49–51]. GE11 conjugation has been shown to enhance tumor‐specific internalization and gene expression in EGFR‐overexpressing tumors while maintaining low mitogenicity. Similar targeting strategies in other carriers improve tumor accumulation, uptake, and therapeutic efficacy while reducing off‐target effects [52, 53]. Integrating GE11 with GO‐based multigene carriers provides a rational strategy for selective and potent reprogramming of the tumor microenvironment, maximizing cancer cell elimination while minimizing unintended effects on healthy tissues.

## 2. Materials and Methods

### 2.1. Cell Culture

Human lung carcinoma A549 cells (ATCC, USA) were maintained in Dulbecco’s modified Eagle medium (DMEM, Gibco, USA) supplemented with 10% fetal bovine serum (FBS, Gibco). Human lung fibroblasts (HLF; Lonza, Canada) were cultured in fibroblast growth basal medium (FBM‐2) supplemented with 5% Insulin, 5% human fibroblast growth factor (hFGF‐B), and 5% gentamicin sulfate‐amphotericin (GA‐1000; FGM‐2 Fibroblast Growth Medium‐2 BulletKit, Lonza, Canada). THP1 monocytes (ATCC, USA) were maintained in RPMI‐1640 medium (Gibco) containing 10% FBS and 2‐mercaptoethanol to a final concentration of 0.05 mM. DCs (ABM, Canada) were maintained in PriGrow X Series Medium for T0525 (ABM, Canada) supplemented with 10% FBS. All cells were incubated at 37°C in a humidified atmosphere with 5% CO_2_.

### 2.2. Establishment of the 3D Lung Cancer Model

A 3D lung cancer model containing A549 cancer cells (A), HLF fibroblasts (F), and THP1 (T) was developed following the same procedure as described in our previous work (referred to as the AFT model) [37]. Briefly, to prepare the ECM, Matrigel Matrix Basement Membrane Growth factor reduced (Corning, USA) was mixed with collagen type I (3 mg/mL, Corning) in a 1:1 ratio on ice. The cell mixture (A:F:T, 1:1:1, 15 × 10^3^ cells/model) was resuspended in the Matrigel‐collagen solution and deposited as droplets (5 μL) into Nunclon Sphera low‐attachment well plates (Thermo Fisher Scientific, Canada). After polymerization at 37°C for 1 h, prewarmed culture medium (DMEM with 10% FBS) was added gently to each well. The constructs were maintained for 6 days to allow complete cellular organization within the 3D matrix before any treatment. AFTs were treated with 50 ng/mL of Phorbol 12‐myristate 13‐acetate (PMA, Sigma‐Aldrich, Canada) for THP1 macrophage activation. After 24 h of PMA treatment, the medium was replaced with fresh medium, and the cells were allowed to recover for 24 h prior to further treatments.

Control models containing two cell lines (A549 and HLF: AF model; A549 and THP1: AT model; HLF and THP1: FT model) were realized following the same procedure. For immune‐specific control experiments, additional models incorporating DCs were prepared. AF‐DC constructs (A:F:DC, 1:1:1; 15 × 10^3^ cells per model) and AFT‐DC constructs (A:F:T:DC, 1:1:0.5:0.5; 15 × 10^3^ cells per model) were generated using the same matrix preparation and culture protocol.

### 2.3. Functionalization of GO Nanocarriers

GO sheets were produced from natural graphite powder following a modified Hummers’ oxidation method, as previously reported [33, 34]. The oxidized product was repeatedly washed and filtered to obtain a single‐layer GO with lateral dimensions exceeding 650 nm. The resulting dispersion was thoroughly rinsed with deionized water and stored at 1 mg/mL. To generate the small‐sized formulation, GO sheets were subjected to controlled intermittent probe sonication (probe sonicator, Cole Parmer, Canada) at low power (10 W) and 0°C to obtain nanosheets with an average lateral size of approximately 100 nm [33, 34].

GO was functionalized with six‐arm amine‐terminated PEG (15 kDa; Jenkem Technology, USA) and fourth‐generation, primary amine polyamidoamine (PAMAM, 10 kDa; 10.1 w/w% in water, Dendritech Inc., USA) using carbodiimide coupling chemistry, as previously described [33]. In our previous study, FTIR analyses were conducted to validate the effectiveness of surface functionalization of GO flakes with PEG and PAMAM. AFM and DLS measurements were also used to determine the morphology, dispersion, and size distribution of GO‐PEG‐PAMAM nanocarriers. Detailed results can be found in our previous publications [33, 39]. To confirm the consistency of the nanocarrier formulation used in the present study, DLS measurements were performed on the modified GO flakes dispersed in Milli‐Q water (5 μg/mL) using a Zetasizer Nano ZS (Malvern Instruments, UK) at 25°C following 180‐s equilibration (Figure S1 and Table S1). The nanocarriers exhibited a Z‐average hydrodynamic diameter of 221 ± 2.4 nm and a polydispersity index (PDI) of 0.205 ± 0.008 (mean ± standard deviation [SD], *n* = 5 independent measurements). The approximately 118 nm value reported in our previous work corresponds to the number‐weighted particle diameter obtained by DLS, which closely reflected the AFM‐derived lateral dimensions (100 nm) of the small GO nanosheets [33]. In contrast, the present study reports the Z‐average hydrodynamic diameter (221 ± 2.4 nm), an intensity‐weighted cumulants parameter that is more sensitive to the hydrated polymer coating and the presence of larger particles or minor aggregates in suspension. Consequently, the Z‐average is expected to exceed the previously reported number‐weighted diameter, and these measurements should not be compared directly. For experiments involving targeted nanocarriers, the GO‐PEG‐PAMAM material was biofunctionalized by further conjugation with the GE11 peptide (amino acid sequence YHWYGYTPQNVI, MW ≈ 1757 Da; GenScript, USA) via carbodiimide‐mediated coupling. GE11 solution (1 mg/mL) was mixed with 0.1 M MES buffer (pH 5.5) and activated with freshly prepared 1‐ethyl‐3‐(3‐dimethylaminopropyl)carbodiimide hydrochloride (EDC, Sigma‐Aldrich, USA; 0.546 mg, 2.846 μmol) and N‐hydroxysuccinimide (NHS, Sigma‐Aldrich; 0.328 mg, 2.846 μmol) for 20 min at room temperature to form reactive NHS esters. The activated GE11 solution was then added dropwise to the GO‐PEG‐PAMAM dispersion under gentle stirring, and the mixture was incubated at room temperature for 4 h. The resulting GE11‐functionalized nanocarrier was washed repeatedly with water using Amicon Ultra centrifugal filters (30 kDa; MilliporeSigma, USA) to remove unreacted reagents. The final dispersion was stored at 4°C for further use in selected experiments. The Z‐average hydrodynamic diameter of 111.5 ± 2.4 nm and a PDI of 0.175 ± 0.009 (mean ± SD, *n* = 5 independent measurements) were measured for the GO‐GE11 nanocarriers (Figure S1). For simplicity, the untargeted formulation, GO‐PEG‐PAMAM, will be referred to as GO, and the GO‐PEG‐PAMAM‐GE11 formulation as GE11/GO.

For uptake analysis, GO and GE11/GO were labeled with Alexa Fluor 488 or 568 dyes (Conjugation Kit (Fast) Lightning‐Link, Abcam, USA) following the manufacturer’s protocol [37]. Briefly, the modifier was added to 10 μg of GO or GE11/GO (1 mg/mL), mixed with the lyophilized Alexa Fluor conjugation mix, and incubated for 15 min. Finally, the quencher was added, and the mixture was incubated for 5 min. The conjugated material was stored at 4°C in darkness.

### 2.4. Gene Loading and Transfection Procedures

siRNA duplexes targeting CD47 and TGFβ (CD47_siRNA and TGFβ_siRNA; IDT, USA), and plasmid DNA encoding ANT1 (ANT1_pDNA; Origene, USA) were complexed with GO to form transfection mixes. To form the transfection mix GO‐CD47‐ANT1, CD47_siRNA and ANT1_pDNA were first mixed in Opti‐MEM Reduced‐Serum Medium (Gibco) at a 1:1 w/w ratio and subsequently complexed with GO at a total nucleic acid:GO ratio of 3:1 (w/w), corresponding to a final CD47_siRNA:ANT1_pDNA:GO ratio of 1.5:1.5:1 (w/w/w) [31]. For GO‐TGFβ, TGFβ_siRNA was complexed with GO at a TGFβ_siRNA:GO ratio of 1.5:1 (w/w). Mixtures were incubated for 60 min at room temperature to enable electrostatic adsorption of nucleic acids onto the nanocarrier. The loading of nucleic acids onto GO nanocarriers has been previously evaluated using complementary approaches. In particular, the loading efficiency of CD47_siRNA onto small modified graphene oxide (smGO) carriers (the same GO nanocarrier platform used in the present study) was assessed by monitoring absorbance at 260 nm as a function of increasing siRNA:GO binding ratios (w/w 1−10), demonstrating enhanced signal with increasing complexation [33]. In addition, successful gene loading was confirmed by zeta potential measurements, which showed a shift in surface charge from positive values (around +18 mV) to approximately −18 to −20 mV following complex formation, consistent with electrostatic binding [25].

Before transfection, the culture medium was removed and replaced with Opti‐MEM Reduced Serum Medium without serum. GO‐CD47‐ANT1 or GO‐TGFβ formulations were then added to AFT constructs at a final GO concentration of 0.5 μg/mL, and the constructs were maintained in this medium for 72 h under standard incubation conditions. For combinatorial transfection, GO‐CD47‐ANT1 and GO‐TGFβ were administered simultaneously at a 1:1 mass ratio, resulting in a total GO concentration of 1 μg/mL. Control samples included untreated AFT models (untransfected control), AFT models treated with naked nucleic acids (i.e., CD47_siRNA, ANT1_pDNA, TGFβ_siRNA), and GO flakes without genes. Transfection efficiency and gene modulation were analyzed at 72 h post‐treatment. Gene loading and transfection procedures using GE11/GO were performed following the same methods.

### 2.5. Immunohistochemistry (IHC)

For IHC, AFT were washed with phosphate‐buffered saline (PBS) (Thermo Fisher Scientific, Canada) and fresh‐frozen in optimal cutting temperature (OCT) compound (Fisher Healthcare Tissue‐Plus, Thermo Fisher Scientific, Canada) to generate tissue blocks. The frozen blocks were used to prepare frozen sections (10 μm thick), which were placed on glass slides. The sections were then washed with PBS, permeabilized using 0.1% Triton X‐100 (Sigma‐Aldrich, USA), and blocked in 5% normal goat serum (Thermo Fisher Scientific, Canada) for 1 h at RT. Primary antibodies against Caspase 9, cluster of differentiation 68 (CD68), E‐cadherin (Thermo Fisher Scientific, Canada), alpha‐smooth muscle actin (αSMA), vimentin, and cytokeratin 7 (CK7; Abcam) were applied overnight at 4°C. After washing, Alexa Fluor 488‐ or 555‐conjugated goat anti‐rabbit or anti‐mouse secondary antibodies (Thermo Fisher Scientific, Canada) were added for 1 h at room temperature. Nuclei were counterstained with Hoechst 33342 (Thermo Fisher Scientific, Canada), and samples were mounted for imaging using Prolong Gold Antifade Mountant (Thermo Fisher Scientific, Canada) and cured overnight before visualization.

Fluorescence images were acquired with an Echo Revolve R4 (Discover Echo, USA) microscope equipped with 10× magnification objective (Olympus Life Science, Japan), and DAPI (ex: 380/30; em: 450/50), FITC (ex: 470/40; em: 525/50), and TRITC (ex: 530/40; em: 605/70) filters. The images (895 × 750 μm^2^ and 2448 × 2048 pixel^2^) were analyzed using ImageJ [54]. For image intensity quantifications, fluorescence signals were measured across the entire image area after background correction using cell‐free regions. Background‐subtracted integrated fluorescence intensity of the target protein was determined for each section and divided by the corresponding nuclei count to account for differences in cell number. Protein expression levels were then normalized to the untransfected control and expressed as relative fold change.

For cell counting and co‐localization analyses, fluorescence thresholds were manually established from the corresponding control images to distinguish positive staining from background signal. The same threshold values were subsequently applied to all images within the same experiment. Individual cells were identified by nuclear staining, and co‐localization of the target signal with CK7 staining was assessed on a per‐cell basis. The numbers of double‐positive, single‐positive, and double‐negative cells were expressed as percentages of the total number of cells in each population (CK7^+^ or CK7^−^).

Three sections of three different spheres (three technical replicates) from three separate experiments (three biological repeats) to varying depths within the center of the AFT model were used for quantifications.

### 2.6. RT‐qPCR

Total RNA was extracted from the 3D AFT constructs using the RNeasy Micro Kit (Qiagen, USA) following the manufacturer’s protocol. RNA purity and concentration were measured with a NanoDrop 2000 spectrophotometer (Thermo Fisher). RT‐qPCR was performed on an Applied Biosystems 7500 Fast Real‐Time PCR System, and reverse transcription and amplification were performed with the Power SYBR Green RNA‐to‐CT 1‐Step Kit (Thermo Fisher) using gene‐specific primers for the housekeeping gene GAPDH and the targets CD47, ANT1, TGFβ, CD68, IL1β, TNFα, IL6, CD163, and IL10 (Quantitect Primer assays, Qiagen, Canada). qPCR reactions were conducted in 96‐well optical plates (Applied Biosystems, USA) in triplicate. Relative expression levels were determined using the 2^−ΔΔCt^ method. Briefly, gene expression was normalized to the housekeeping gene GAPDH to obtain the ΔCq value for each target, which was then averaged across triplicates and normalized to the untransfected control to obtain the ΔΔCq. An exponential transformation (ΔΔCq expression = 2^−ΔΔCq^) was used to obtain the fold change for each target relative to the untransfected control. Amplification specificity was verified by melt curve analysis following each qPCR run. Representative melt curves for all primer pairs are provided in the Supporting Information (Figure S2).

### 2.7. Cell Viability Evaluation

Cell viability was determined using the Cell Titer‐Glo Luminescent Cell Viability Assay (Promega, USA), and cell damage was assessed using the Lactate Dehydrogenase (LDH) Assay kit (Abcam, Canada) according to the manufacturer’s instructions. For viability analysis, AFT constructs were transferred into opaque white 96‐well plates (Corning) and equilibrated to room temperature before reagent addition (1:1 w/w reagent‐to‐medium). For ATP extraction, plates were shaken at 800 rpm for 10 min to ensure effective extraction of ATP from the cells, then incubated at room temperature for 30 min at 500 rpm. Luminescence was recorded with a FLUOstar Omega microplate reader (Mandel Scientific, Canada). For LDH assessment, 100 μL of the LDH reaction mix was added to 10 μL of media in a transparent 96‐well plate, and the mixture was incubated for 30 min at RT before absorbance was measured at 490 nm. Data were normalized to untransfected controls to determine percentage viability and apoptotic activity.

### 2.8. Statistical Analysis

All data are presented as mean ± SD. RT‐qPCR results are obtained from three biological repetitions (*n* = 3). For immunohistochemical image quantification, three sections per model (at different depths from the central region of the model), three technical replicates, and three biological replicates (*n* = 3) were analyzed. The results were averaged to obtain the final values and SD. Microplate reader results are given by three biological repetitions (*n* = 3) of a minimum of three technical replicates. All statistical analyses were performed with GraphPad Prism 8.02 (GraphPad Software, USA). Variations across more than three conditions were analyzed using a one‐way ANOVA. Two‐way ANOVA was used to assess the effects of two independent variables on a dependent variable. When ANOVA identified significant differences, post hoc multiple‐comparison tests were performed using either Tukey’s test for all pairwise comparisons or Dunnett’s test when comparing treatment groups against a single control group. A value of *p* < 0.05 was considered statistically significant.

## 3. Results and Discussion

### 3.1. 3D Multicellular Cancer Model Recapitulates Tumor‐Immune‐Stroma Interactions

**FIGURE 1:**
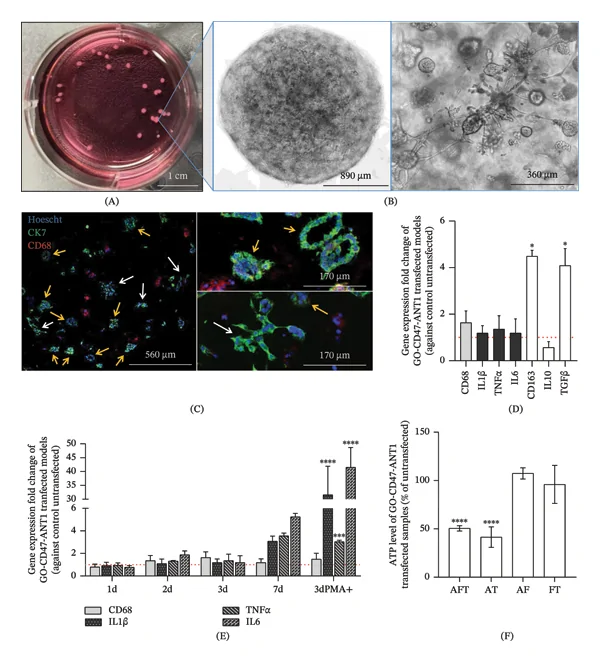
Characterization of the AFT 3D lung cancer model and macrophage polarization dynamics. (A) AFT model in a multiwell after 6 days of incubation. Scale bar = 1 cm. (B) Bright‐field image showing the entire structure (left panel) and internal 3D cellular organization (right panel). Scale bars = 890 and 360 μm. (C) IHC images of 10 μm thick sections from the central region of the AFT model. A low‐magnification overview is shown on the left panel, while the right panel shows higher‐magnification views. Blue: Hoechst nuclei; green: CK7^+^ cancer cells; red: CD68^+^ THP1‐derived macrophages. CK7^+^ cells form spheroidal‐like constructs (orange arrows) and disorganized structures (white arrows). Scale bars = 560 and 170 μm. (D) qPCR of macrophage polarization cytokines and markers after 72 h GO‐CD47‐ANT1 transfection. Bar graph displays M0 (CD68), M1 (IL1β, TNFα, IL6), and M2 (CD163, IL10, TGFβ) gene expression. (E) qPCR analysis over different transfection times and comparison with 3‐day‐transfected PMA‐stimulated AFT constructs. (F) Cell viability (ATP quantification) in AFT and control co‐culture models (AT: A549&THP1, AF: A549&fibroblasts, FT: fibroblasts&THP1) after GO‐CD47‐ANT1 transfection, highlighting PMA effects. Statistical significance was determined against the untransfected AFT model using one‐way and two‐way ANOVA (^∗^ *p* < 0.05; ^∗∗^ *p* < 0.01; ^∗∗∗^ *p* < 0.001; ^∗∗∗∗^ *p* < 0.0001; and *p* > 0.05, not significant, not shown).

To enable mechanistic analysis of macrophage differentiation, polarization, and therapeutic response within a lung tumor microenvironment, we established 3D constructs composed of A549 lung cancer cells, lung fibroblasts, and THP1 monocytes, as previously described by our group [37]. Single‐cell suspensions of A549, fibroblasts, and THP1 cells (AFT model) were encapsulated within Matrigel‐collagen microdroplets to support 3D growth and spontaneous cell–cell and cell–matrix interactions (Figure 1A). Although the employment of a single lung cancer cell line does not fully capture the intratumoral heterogeneity characteristic of clinical tumors, A549 cells were selected because they represent a well‐characterized lung adenocarcinoma model that exhibits robust growth in 3D culture and has been extensively used in studies of therapeutic resistance, tumor–microenvironment interactions, and targeted gene delivery, offering a controlled and reproducible system for evaluating therapeutic strategies [55]. The encapsulated cells self‐assembled into integrated tumor‐stroma‐immune spheroids with a spherical morphology and an average diameter of 1972 ± 80 μm (Figure 1B). This value was calculated from measurements of 30 spheroids obtained across three independent biological replicates (10 spheroids per replicate) and falls within a size range that enables the development of spatially heterogeneous microenvironments and immune‐stromal interactions within the spheroid. Although it remains a microscale construct and does not fully recapitulate the vascular heterogeneity, perfusion dynamics, and transport barriers present in established tumors in vivo, its size is sufficient to generate spatially heterogeneous cellular organization and diffusion gradients while maintaining experimental reproducibility and accessibility for mechanistic studies. By day 11, CK7^+^ lung adenocarcinoma [11, 56] cancer cells constituted 77 ± 2% of the total population (Figure S3) and organized into cohesive, spheroid‐like epithelial structures (Figure 1C, orange arrows), a characteristic feature of epithelial tumor dominance in 3D matrices that reflects strong cell–cell adhesion and coordinated tissue organization [11, 21, 57].

THP1 monocyte differentiation into macrophages was confirmed by dense CD68^+^ multicellular clusters [24, 58], accounting for 16 ± 2% of the population (Figure 1C and Figure S3) [9, 18]. Lung fibroblasts (CK7^−^/CD68^−^) represented 7 ± 0.2% of the population and exhibited slower proliferation and a dispersed spatial distribution (Figure 1C and Figure S3), consistent with their supportive yet regulatory role in 3D tumor architecture rather than direct tissue dominance [9, 16, 21].

After 6 days of maturation, AFT constructs were treated with GO‐CD47‐ANT1 nanocarriers, a gene‐delivery system previously validated by our group for efficient modulation of immune evasion and apoptotic signaling in cancer cells [37]. Transcriptional profiles of macrophage‐associated cytokines and polarization markers associated with pro‐inflammatory (M1‐like) or immunosuppressive, tissue‐remodeling (M2‐like) phenotypes were then analyzed. The panel included M1‐associated cytokine genes (IL1β, TNFα, and IL6) and M2‐associated markers (CD163, IL10, and TGFβ), which are commonly linked to inflammatory versus immunosuppressive, extracellular‐matrix‐remodeling macrophage states [9, 24, 58].

Gene expression analysis in Figure 1D showed that macrophages adopted a predominantly immunosuppressive transcriptional profile following GO‐CD47‐ANT1 transfection, with mRNA levels of CD163 and TGFβ increasing 4.5‐fold and 4.1‐fold, respectively, and only minimal induction of IL1β, TNFα, and IL6 (∼1.2–1.4‐fold). Although IL10 transcripts were reduced (∼0.6‐fold), the overall transcriptional profile, characterized by increased CD163 and TGFβ, indicates a shift toward M2‐like tissue‐remodeling and tumor‐supportive macrophage states, reflecting transcriptional activity rather than absolute cytokine abundance [25]. This discrepancy suggests that 3D tumor microenvironment actively dampens macrophage inflammatory activation, even in the presence of gene‐delivery interventions, likely through sustained cancer‐fibroblast‐macrophage crosstalk that reinforces M2‐like polarization cues. These results are in line with the 3D tumor‐immune‐fibroblast models, which show that concurrent cancer–fibroblast interactions are required to promote CD163^+^ M2‐like macrophages and that THP1 cell polarization depends on interactions with other cell types [9].

We next monitored IL1β, TNFα and IL6 transcript levels from 1 day to 7 days after transfection (Figure 1E). The gradual increase in M1 cytokine transcripts over 7 days indicates delayed immune activation, consistent with progressive relief of tumor‐mediated immunosuppression rather than immediate inflammatory reprogramming. Exposure to PMA, a common treatment for differentiating THP1 cells into macrophages prior to transfection [58, 59], led to a rapid and robust increase in M1 cytokine mRNA levels within 72 h of transfection (Figure 1E), with IL1β, TNFα, and IL6 transcript levels increasing by 31.6‐fold, 3‐fold, and 41.5‐fold, respectively. Consistent with known PMA effects on THP1 differentiation, these results demonstrate that macrophage inflammatory activation is intrinsically suppressed within the 3D tumor microenvironment and requires strong exogenous pro‐inflammatory cues for rapid induction [59].

A pro‐inflammatory macrophage phenotype is usually associated with boosted cytotoxic immune functions, increased phagocytosis of malignant cells, and restriction of cancer cell growth and invasion [58]. Consequently, given the enhanced pro‐inflammatory environment induced by pretreating AFT models with PMA, we then analyzed the cell viability after transfection. Figure 1F shows that AFT viability dropped to around 51%. AF models lacking immune cells exhibited no measurable reduction in viability, suggesting that macrophages contribute to the observed therapeutic response and cancer cell elimination. This interpretation is supported by previous co‐culture studies, in which modulation of CD47‐calreticulin axis was directly associated with macrophage‐dependent elimination of cancer cells. Notably, the same smGO/CD47_siRNA platform promoted phagocytic elimination of A549 and other cancer cells in macrophage co‐culture [25], while a related stimulated macrophage leukemia model demonstrated a similar relationship between reduced CD47, increased calreticulin, and cancer‐cell clearance [31]. Nevertheless, phagocytosis was not directly quantified in the present 3D AFT model, and macrophage involvement is therefore inferred from the model‐comparison and gene‐expression results.

In contrast, the FT model, which contained no cancer cells, maintained high viability, indicating that the gene‐therapy effects were primarily directed toward cancer cells rather than stromal components. Notably, AT models containing cancer and immune cells but lacking the fibroblasts exhibited higher sensitivity, with a reduced cell viability to 41%. These findings demonstrate that fibroblasts exert a dominant protective effect against gene‐therapy‐induced cytotoxicity, substantially reducing overall treatment sensitivity. This reduced response aligns with extensive evidence implicating fibroblasts in therapy resistance through ECM deposition, immune exclusion, and impaired therapeutic penetration [60–62].

The observed delayed M1 cytokine induction highlights the intrinsic immunosuppressive nature of the 3D tumor microenvironment, consistent with in vivo observations where macrophage activation is constrained by tumor and stromal signaling. Fibroblasts, representing only ∼7% of the population, exerted a disproportionately strong protective effect, reflecting their known roles in ECM deposition, immune exclusion, and physical barrier formation that limit therapeutic penetration [60–62]. These results highlight the importance of incorporating stromal components into preclinical models, as their absence may lead to an overestimation of treatment efficacy. Furthermore, the gradual increase in IL1β, TNFα, and IL6 over 7 days suggests that the timing of gene delivery and therapeutic readouts should account for the progressive relief of tumor‐mediated immunosuppression, which may be critical for optimizing combination interventions. Altogether, these results establish that although pro‐inflammatory macrophage activation enhances therapeutic efficacy, stromal components—particularly fibroblasts—remain dominant regulators of treatment resistance. This interplay motivates strategies that combine immune activation with targeted stromal reprogramming, as described in subsequent sections.

### 3.2. TGFβ Targeting Reprograms Stromal and Immune Features

#### 3.2.1. Optimization of Multigene Delivery in the AFT Model

**FIGURE 2:**
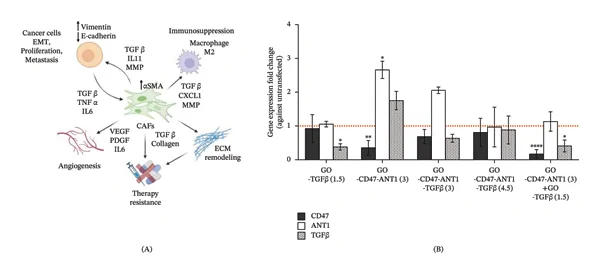
GO‐based nanocarriers enable multigene regulation of TGFβ, CD47, and ANT1. (A) Schematic representation of the interactions among cancer cells, fibroblasts, and macrophages mediated by TGFβ within the tumor microenvironment. The diagram summarizes known pathways involved in stromal activation and cancer‐supportive functions mediated by CAFs. (B) qPCR measurements of CD47, ANT1, and TGFβ gene expression regulation following transfection with different formulations of GO‐based delivery systems. Statistical significance was determined against the untransfected AFT model using two‐way ANOVA, with ^∗^ *p* < 0.05; ^∗∗^ *p* < 0.01; and ^∗∗∗∗^ *p* < 0.0001 considered significant, and *p* > 0.05 was considered not significant.

In tumor contexts, fibroblasts readily transition into CAFs, adopting a contractile and highly secretory phenotype that amplifies multiple tumor‐supportive processes. As schematically illustrated in Figure 2A, activated CAFs enhance cancer cell aggressiveness by promoting epithelial–mesenchymal transition (EMT) through the secretion of cytokines, chemokines, and matrix‐modifying enzymes [12, 16, 20, 21]. At the immune level, CAFs secrete mediators such as TGFβ that recruit and polarize macrophages toward an immunosuppressive, M2‐like phenotype, thereby weakening local antitumor immunity [9, 42].

CAFs further drive extensive ECM remodeling through collagen‐rich matrix deposition, facilitating cancer cell migration while creating physical barriers to therapeutic penetration [12]. Disrupting this CAF‐centered signaling loop can attenuate reciprocal crosstalk between stromal, immune, and cancer cells, thereby constraining tumor progression.

Central to all these interconnected stromal and immune functions is TGFβ, which acts as a dominant upstream regulator of CAF activation, immune suppression, and therapy resistance [46]. Selective inhibition of TGFβ1 has been shown to alleviate resistance to immune checkpoint blockade by reshaping the tumor immune microenvironment [63]. Given its pivotal role in cancer support, we sought to incorporate TGFβ_siRNA into our therapeutic strategy to enable TGFβ silencing.

To identify an effective co‐delivery strategy for simultaneous modulation of CD47, ANT1, and TGFβ expression, multiple loading configurations and GO:siRNA:pDNA ratios were evaluated based on mRNA fold‐change outcomes (Figure 2B). Treatment with GO‐TGFβ_1.5, corresponding to a GO:siRNA ratio of 1:1.5 (w/w), produced a substantial and specific reduction of TGFβ mRNA levels (0.4‐fold) without altering CD47 or ANT1 gene expression. In contrast, the GO‐CD47‐ANT1_3 formulation (1:3, GO:nucleic acid, w/w, with 1:1 siRNA:pDNA, w/w) efficiently downregulated CD47 transcripts (0.4‐fold) while inducing ANT1 expression (2.7‐fold), but concomitantly increased TGFβ mRNA levels (1.8‐fold). This upregulation may reflect a compensatory response of the tumor microenvironment to apoptotic stress associated with CD47 silencing and ANT1 overexpression. Previous studies have shown that apoptotic tumor cells can contribute to increased TGFβ signaling, promoting the establishment of an immunosuppressive niche that limits antitumor immune responses, while surviving tumor and stromal cells may also contribute to increased TGFβ production as part of an adaptive tissue remodeling response [64–66]. Together, these responses may attenuate the long‐term efficacy of apoptosis‐inducing therapies. Simultaneous silencing of TGFβ may therefore counteract this compensatory feedback, providing a mechanistic rationale for the enhanced therapeutic efficacy observed with the combined GO‐CD47‐ANT1 and GO‐TGFβ treatment.

When all three nucleic acids were co‐loaded onto a single nanocarrier, the GO‐CD47‐ANT1‐TGFβ_3 formulation (1:3, GO:nucleic acids, with 1:1:1 siRNA:pDNA:siRNA, w/w/w) produced only partial and nonsignificant modulation of each target gene (CD47: 0.7‐fold; ANT1: 2.1‐fold; TGF‐β: 0.6‐fold). The reduced transcriptional modulation relative to the GO‐TGFβ and GO‐CD47‐ANT1 formulations suggests a lower overall transfection efficiency, likely due to partial competition among the three nucleic acids for GO binding sites.

Notably, in our previous work [33], systematic GO‐CD47_siRNA binding studies revealed a progressive increase in absorbance with increasing siRNA loading until saturation between ∼1:3 and 1:5 (GO:siRNA, w/w). This saturation behavior reflects the nanocarrier’s finite loading capacity; additional nucleic acid beyond this threshold results in steric crowding and reduced adsorption efficiency [36, 67]. This is particularly relevant for multigene loading, because siRNAs and pDNAs differ markedly in size, steric hindrance, charge distribution, and structural flexibility. siRNAs are short and rigid, whereas pDNA is much larger and requires substantially more surface area for condensation [68]. Once the GO surface approaches saturation, competition among cargos becomes pronounced, destabilizing the complex and reducing reproducibility, consistent with the attenuated gene modulation observed for the triloaded formulation. This interpretation is further supported by the GO‐CD47‐ANT1‐TGFβ_4.5 formulation (1:4.5, GO:nucleic acids, with 1:1:1 siRNA:pDNA:siRNA, w/w/w), which exhibited weaker and more variable transcriptional modulation across all three targets, consistent with excessive payload competition (CD47: 0.8‐fold; ANT1: 1.0‐fold; TGFβ: 0.9‐fold).

To overcome payload competition and surface saturation observed in triloaded GO systems, a two‐system co‐delivery strategy was evaluated by combining the GO‐CD47‐ANT1_3 formulation (1:3, GO:nucleic acids, with 1:1 siRNA:pDNA, w/w) with the GO‐TGFβ_1.5 system (1:1.5; GO:siRNA, w/w). This two‐GO carrier strategy produced robust CD47 knockdown (0.2‐fold) and efficient TGFβ silencing (0.4‐fold); however, ANT1 induction was reduced relative to that observed with the single GO‐CD47‐ANT1 system. The reduced ANT1 induction is likely influenced by early cancer cell loss induced by simultaneous CD47 and TGFβ suppression, which decreases the number of viable cancer cells contributing to bulk transcript measurements at the selected time point. This interpretation is further supported by the overall reduction in CD47 and TGFβ transcript abundance relative to single‐system transfections, consistent with a decreased population of viable, transcriptionally active cancer cells rather than reduced delivery efficiency. Overall, these results suggest that the optimal time point for capturing ANT1 overexpression following combined GO‐CD47‐ANT1_GO‐TGFβ treatment likely occurs earlier, prior to the onset of extensive apoptosis. This emphasizes the necessity of temporal profiling in multigene strategies to capture transient expression dynamics that may otherwise underestimate therapeutic impact.

#### 3.2.2. TGFβ Silencing Modulates CAF Phenotypes and Stromal Signaling

**FIGURE 3:**
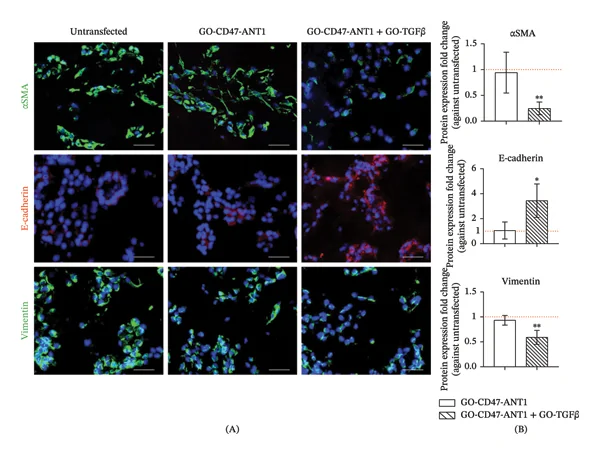
TGFβ implication on fibroblast activation in the AFT model. (A) IHC images of AFT model sections (10 μm thick) showing expression of αSMA (green), vimentin (green), and E‐cadherin (red) in untransfected, GO‐CD47‐ANT1‐transfected, and GO‐CD47‐ANT1_GO‐TGFβ‐transfected constructs. Nuclei are identified by Hoechst staining (blue). Scale bar: 50 μm. (B) Quantification of fluorescence intensity for αSMA, vimentin, and E‐cadherin across treatment conditions relative to the untransfected control. Statistical significance was determined against the untransfected AFT model using one‐way ANOVA, with ^∗^ *p* < 0.05 and ^∗∗^ *p* < 0.01 considered significant, and *p* > 0.05 was considered not significant.

Having established a delivery strategy that balances loading efficiency with biological timing, we next evaluated how concurrent TGFβ silencing alters CAF activation, ECM organization, and macrophage polarization within the 3D AFT model. TGFβ silencing is expected to reprogram CAFs from a contractile, ECM‐producing, and immunosuppressive state toward a quiescent, less tumor‐supportive phenotype, an effect that we next confirmed in the AFT model. Immunofluorescence of untransfected AFT constructs (Figure 3A) confirmed robust αSMA and vimentin expression, indicating fibroblast transition into CAFs and an activated stromal compartment capable of ECM remodeling and promoting cancer cell motility [20, 21]. Concurrently, low E‐cadherin expression in cancer cells confirmed ongoing EMT, consistent with TGFβ‐driven stromal signaling and acquisition of a more invasive phenotype [45]. Only the combined GO‐CD47‐ANT1_GO‐TGFβ treatment significantly modulated these markers, reducing αSMA and vimentin to 0.2‐fold and 0.6‐fold, respectively, and restoring E‐cadherin 3.4‐fold (Figure 3B), consistent with CAF deactivation and partial reversal of EMT in cancer cells. No such changes were observed with GO‐CD47‐ANT1 alone, indicating that CD47/ANT1 modulation does not affect CAF activation or epithelial integrity. In contrast, TGFβ silencing reduces CAF and mesenchymal features, confirming that TGFβ is a critical regulator of stromal‐driven tumor support. These results are consistent with previous reports showing that TGFβ inhibition in stroma‐rich models reduces αSMA expression and ECM deposition, facilitating improved therapeutic penetration [45, 46, 48]. In the AFT model, TGFβ silencing reduced αSMA and vimentin expression while partially restoring E‐cadherin expression, consistent with attenuation of CAF‐associated features and partial EMT reversal, and coincided with enhanced sensitivity to CD47/ANT1‐mediated cytotoxicity achieved with the GO‐based tridelivery system. Collectively, these stromal and epithelial changes correlate with reduced cancer cell invasion and enhanced therapeutic sensitivity, emphasizing the central role of TGFβ in maintaining a tumor‐supportive microenvironment.

In alignment with previous studies showing that stroma‐targeted interventions promote immune‐cell infiltration and drug delivery [45, 46, 48], these findings support a dual‐modulation paradigm in which stromal normalization primes the microenvironment for targeted cancer cell elimination. In addition to remodeling the stroma, TGFβ inhibition shifts macrophage polarization toward an M1‐dominant, pro‐inflammatory state. It acts synergistically with CD47 silencing and ANT1 expression to augment macrophage activation and tumor‐cell recognition.

#### 3.2.3. TGFβ Silencing Biased Monocyte Polarization Toward a Pro‐Inflammatory, M1‐Like Phenotype

**FIGURE 4:**
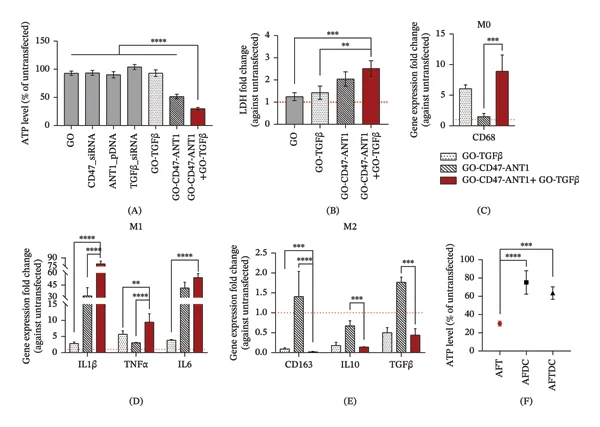
TGFβ involvement in macrophage activation and cancer cell elimination. (A) Cell viability (ATP quantification) and (B) cell death (LDH release) in AFT models treated with GO‐TGFβ, GO‐CD47‐ANT1, or the combined GO‐CD47‐ANT1_GO‐TGFβ therapy. qPCR measurements of genes expressing for (C) CD68, (D) M1‐associated (IL1β, TNFα, IL6), and (E) M2‐associated proteins (CD163, IL10, TGFβ) following transfection with different GO‐based gene‐delivery formulations. (F) Cell viability (ATP quantification) in AFT and control models containing THP1 and/or DC following treatment with the combined GO‐CD47‐ANT1_GO‐TGFβ system. Statistical significance was assessed relative to the AFT model treated with GO‐CD47‐ANT1_GO‐TGFβ using one‐ or two‐way ANOVA, with ^∗^ *p* < 0.05; ^∗∗^ *p* < 0.01; ^∗∗∗^ *p* < 0.001; and ^∗∗∗∗^ *p* < 0.0001 considered significant, and *p* > 0.05 was considered not significant (not shown).

Having demonstrated that TGFβ signaling regulates CAF‐associated features and epithelial–mesenchymal plasticity in cancer cells, we next investigated its impact on macrophage phenotype. As an initial step, cell viability and LDH release were assessed to establish baseline cytotoxic effects across the different co‐delivery strategies. Figure 4A,B summarizes the impacts of GO‐CD47‐ANT1_GO‐TGFβ on cell viability and cell death relative to individual GO‐CD47‐ANT1 or GO‐TGFβ treatments.

In untransfected controls and constructs treated with GO alone (without nucleic acids) or with free siRNA/pDNA (without GO), cell viability remained above 90% over 72 h (Figure 4A), confirming baseline biocompatibility [38]. Treatment with GO‐TGFβ alone did not reduce overall viability, indicating that TGFβ silencing primarily modulates the microenvironment rather than directly inducing cancer cell death. In contrast, GO‐CD47‐ANT1 reduced viability to ∼51%, consistent with the combined CD47 and ANT1 formulation [37]. The greater therapeutic response observed in macrophage‐containing models, together with our previous co‐culture studies directly demonstrating CD47‐associated phagocytic cancer cell elimination [25, 31], supports macrophage involvement in the present system. However, phagocytosis and ANT1‐dependent mitochondrial activity were not directly measured in the current study. Importantly, combining GO‐CD47‐ANT1 with GO‐TGFβ further decreased viability to ∼29%, demonstrating that TGFβ silencing sensitizes tumor cells to immune‐ and apoptosis‐mediated killing (Figure 4A).

LDH release (Figure 4B) confirmed these effects: GO‐TGFβ induced minimal LDH release, GO‐CD47‐ANT1 increased LDH 2‐fold, and the combined treatment further elevated it to ∼2.5‐fold. Together, these data indicate that TGFβ silencing enhances the cytotoxic effects of GO‐CD47‐ANT1, consistent with enhanced membrane damage and increased cancer cell susceptibility within the remodeled tumor microenvironment.

Consistent with prior studies, TGFβ silencing alone exerts minimal direct cytotoxicity while sensitizing tumors to immune‐mediated and cytotoxic interventions [43]. In breast and pancreatic cancer models, it deactivates CAFs and normalizes stroma without directly affecting tumor‐cell viability, yet enhances therapeutic responses when combined with cytotoxic or immunomodulatory strategies [46, 69, 70]. These observations align with the evidence that endogenous TGFβ signaling protects tumor cells from therapy‐induced stress. In contrast, its inhibition potentiates apoptosis and limits tumor progression when paired with additional antitumor modalities [48]. In a breast cancer model, a bifunctional therapeutic strategy of combining TGFβ inhibition with immune checkpoint blockade (i.e., anti‐PD‐L1) effectively relieved TGFβ‐driven immune suppression, enabling robust immune‐cell‐dependent tumor regression and prolonged survival [46]. Similarly, in our work, CD47 downregulation has been shown to facilitate cancer cell recognition by macrophages, potentially promoting phagocytic clearance, as previously demonstrated in 2D co‐culture systems [25].

Given the established immunomodulatory role of TGFβ, macrophage‐associated gene expression was next examined to determine whether the observed cytotoxic effects reflected changes in macrophage activation state. CD68 transcripts (Figure 4C and Figure S4) increased 6.1‐fold with GO‐TGFβ and 8.9‐fold with combined GO‐CD47‐ANT1_GO‐TGFβ, whereas GO‐CD47‐ANT1 alone induced a modest 1.5‐fold increase, indicating that TGFβ silencing promotes macrophage differentiation, which is amplified when combined with CD47 blockade.

Analysis of macrophage polarization markers revealed treatment‐dependent polarization profiles (Figure 4D,E and Figure S4). GO‐TGFβ alone moderately upregulated IL1β, TNFα, and IL6 (2.8‐fold, 5.7‐fold, and 3.9‐fold, respectively), reflecting partial macrophage activation upon relief of TGFβ‐mediated immunosuppression (Figure 4D). GO‐CD47‐ANT1 treatment strongly induced IL1β (31.6‐fold), TNFα (3‐fold), and IL6 (41.5‐fold), consistent with M1‐like polarization driven by macrophage engagement with cancer cells. Notably, the combined treatment produced the most pronounced pro‐inflammatory response (IL1β 79‐fold, TNFα 9.5‐fold, IL6 54.4‐fold), indicating synergistic enhancement of M1‐like macrophage activation through concurrent TGFβ inhibition and CD47 blockade.

Concomitantly, expression of M2‐associated markers CD163, IL10, and TGFβ was markedly reduced (0.02‐, 0.1‐, and 0.4‐fold, respectively; Figure 4E and Figure S4), reflecting suppression of immunosuppressive macrophage phenotypes. GO‐CD47‐ANT1 alone moderately upregulated CD163 and TGFβ, indicating partial M2‐like signaling, which was fully counteracted by GO‐TGFβ inclusion.

To directly assess the functional contribution of macrophages, AFT constructs containing THP1 macrophages were compared with models incorporating DCs, a myeloid cell population primarily involved in antigen presentation rather than direct phagocytic killing. In DC‐containing constructs (AF‐DC), the combined GO‐CD47‐ANT1_GO‐TGFβ treatment produced a reduced cytotoxic response (Figure 4F). AF‐DC constructs retained high ATP levels (∼75%), whereas mixed AFT‐DC constructs with fewer THP1 cells showed intermediate viability (∼64%). Importantly, qPCR analysis of gene modulation confirmed that transfection efficiency and target gene regulation were not compromised in DC‐containing models (Table S2), indicating that the reduced cytotoxic outcome is not attributable to impaired delivery or gene silencing. Rather, these findings confirm that macrophage‐specific effector functions are essential for cancer cell elimination, underscoring their central role in the efficacy of GO‐CD47‐ANT1_GO‐TGFβ therapy.

Collectively, these results demonstrate that the therapeutic efficacy of the GO–CD47–ANT1_GO–TGFβ strategy arises from coordinated stromal and immune modulation. TGFβ silencing alleviates CAF‐derived immunosuppressive cues, thereby priming the tumor microenvironment for macrophage activation, while CD47 blockade and ANT1 expression promote tumor‐cell recognition and cytotoxic engagement. Notably, our 3D AFT model demonstrates that robust cytotoxicity requires the presence of THP1 macrophages, confirming that macrophage‐specific effector functions are essential mediators of therapeutic efficacy. This dual modulation paradigm parallels in vivo strategies combining TGFβ pathway inhibition with immune checkpoint blockade, which have consistently shown improved tumor regression compared to single‐agent therapies [35]. Collectively, these results highlight the importance of simultaneously targeting stromal normalization and immune activation to enable effective cancer cell elimination in complex, clinically relevant tumor microenvironments.

### 3.3. EGFR‐Targeted GO‐Genes Delivery Enhances Cancer Cell Specificity

Conventional chemotherapeutic agents often lack cellular specificity, resulting in cytotoxic effects on both malignant and nonmalignant cells within complex tissue models. In 3D co‐culture systems similar to ours, such treatments reduce tumor viability to ∼75% while simultaneously decreasing fibroblast and immune‐cell viability to ∼55% and ∼25%, respectively, underscoring substantial off‐target toxicity [9]. In contrast, targeted therapies have reshaped cancer treatment by improving therapeutic selectivity and limiting collateral damage to healthy tissues [4–8, 71]. In nanomedicine, these benefits arise from preferential cancer cell internalization and stimulus‐responsive payload release. GO is a particularly versatile nanocarrier due to its large surface area and oxygen‐rich functional groups (hydroxyl, carboxyl, and epoxy), which allow extensive surface functionalization and support stimuli‐responsive release mechanisms, including pH‐ and near‐infrared (NIR)‐triggered delivery [35, 36, 67, 72, 73]. The transfection experiments in this study were conducted in Opti‐MEM transfection medium without added serum, and therefore serum‐dependent colloidal stability was not directly assessed. Under future translational conditions, adsorption of serum proteins and protein‐corona formation could alter the hydrodynamic size, surface properties, cellular uptake, and nucleic acid release of the nanocarriers. Polymer functionalization, including PEGylation, has been shown to enhance the dispersion and biological performance of GO‐based gene‐delivery systems [33, 74, 75]; however, the stability of the exact gene‐loaded GO and GE11/GO formulations in serum‐containing media remains to be determined. Together, these features support high drug loading and targeted therapeutic delivery in cancer models.

In our system, while GO‐TGFβ acts broadly within the AFT model to reduce TGFβ‐driven immunosuppressive signaling across stromal and immune compartments, the GO‐CD47‐ANT1 component offers an opportunity for additional refinement toward cancer cell‐selective elimination. To this end, the GO‐CD47‐ANT1 nanocarrier was functionalized with the EGFR‐targeting peptide GE11, enabling preferential delivery of phagocytosis‐ and apoptosis‐regulating genes to EGFR‐expressing cancer cells, while preserving the global microenvironmental modulation mediated by GO‐TGFβ. The impact of this targeting strategy was therefore evaluated by assessing nanocarrier uptake, overall viability, and cancer‐cell‐specific apoptosis within the AFT model.

#### 3.3.1. GE11 Functionalization Improves Uptake

A549 lung adenocarcinoma cells overexpress EGFR, which is widely exploited for targeted delivery. EGFR‐directed ligands enhance nanocarrier uptake in malignant cells while limiting interactions with nonmalignant stromal and immune cells [51, 53].

**FIGURE 5:**
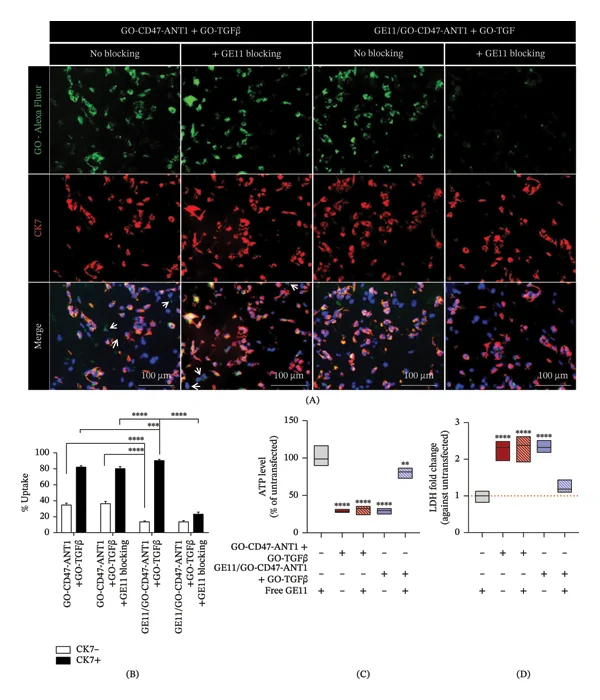
GE11 functionalization enhances cancer‐cell‐specific uptake while preserving therapeutic efficacy. (A) Representative IHC images of AFT sections (10 μm thick) following treatment with Alexa Fluor‐labeled (green) GO‐CD47‐ANT1_ or GE11/GO‐CD47‐ANT1_GO‐TGFβ. CK7 staining (red) identifies cancer cells, and nuclei by Hoechst staining (blue). Controls include unfunctionalized GO‐CD47‐ANT1_GO‐TGFβ and corresponding EGFR‐blocking. White arrows indicate nanocarrier uptake in CK7^−^ cells. Scale bars: 100 μm. (B) Nanocarrier uptake quantified as the percentage of CK7^+^ (cancer) or CK7^−^ (noncancer) cells exhibiting Alexa Fluor signal, normalized to the total number of cells within each population. (C) Cell viability (ATP quantification) of AFT models following treatment with GO‐CD47‐ANT1_GO‐TGFβ and GE11/GO‐CD47‐ANT1_GO‐TGFβ, with and without GE11 blocking. (D) Cell death assessed by LDH release. Statistical significance was determined using one‐ or two‐way ANOVA, with comparisons made relative to the corresponding cell populations in the GE11/GO‐CD47‐ANT1_ GO‐TGFβ‐treated model in panel (B), and to the untransfected control in panels (C) and (D). ^∗∗^ *p* < 0.01; ^∗∗∗^ *p* < 0.001; and ^∗∗∗∗^ *p* < 0.0001 considered significant, and *p* > 0.05 was considered not significant (not shown).

Figure 5A shows representative IHC sections of AFT constructs following treatment with Alexa Fluor‐labeled GO‐CD47‐ANT1 and GE11/GO‐CD47‐ANT1 (used to track the CD47‐ANT1 nanocarrier component; green), together with CK7 staining (red) to identify cancer cells. Both GO‐CD47‐ANT1_GO‐TGFβ and GE11/GO‐CD47‐ANT1_GO‐TGFβ formulations were efficiently internalized, with high uptake observed in CK7^+^ cancer cells. However, qualitative inspection of the sections indicated that nanocarrier internalization by noncancer cells occurred more frequently with the nonfunctionalized formulation (Figure 5A; white arrows), whereas GE11 functionalization visibly reduced off‐target uptake. Image quantification (Figure 5B and Table S3) confirmed these observations. GO‐CD47‐ANT1_GO‐TGFβ was internalized by 82% of CK7^+^ cells and 35% of CK7^−^ cells. GE11/GO‐CD47‐ANT1_GO‐TGFβ increased CK7^+^ uptake to 90% while reducing CK7^−^ uptake to 14%, demonstrating improved cancer cell selectivity primarily through suppression of non‐cancer‐cell internalization.

To determine whether uptake differences were mediated by EGFR‐dependent targeting, blocking experiments were performed by pre‐incubating GE11 prior to transfection. In the absence of GE11 functionalization, blocking did not alter uptake (CK7^+^: 80%; CK7^−^: 36%; Figure 5B and Table S3), consistent with receptor‐independent internalization. In contrast, GE11 blocking strongly reduced uptake of the GE11‐functionalized nanocarrier in CK7^+^ cells (from 90% to 23%; Figure 5B and Table S3), while CK7^−^ uptake remained low and unchanged (∼14%; Figure 5B and Table S3). These results indicate that GE11 functionalization shifts the cancer cell uptake mechanism toward a receptor‐mediated process [49, 52, 53], improving selectivity predominantly by limiting off‐target delivery rather than further increasing absolute cancer cell internalization.

Consistent with these uptake trends, viability (ATP) and cytotoxicity (LDH) measurements confirmed that GE11 functionalization preserves therapeutic efficacy while introducing EGFR‐dependent specificity (Figure 5C,D). Controls treated with GE11 alone and free genes maintained ∼100% viability with no increase in LDH, confirming their lack of intrinsic toxicity under these conditions. GO‐CD47‐ANT1_GO‐TGFβ reduced viability to ∼29% and increased LDH release to ∼2.3‐fold, and this effect was unchanged by GE11 blocking (31% viability; ∼2.3‐fold LDH), supporting a receptor‐independent mechanism for the nonfunctionalized formulation. Similarly, GE11/GO‐CD47‐ANT1_GO‐TGFβ produced cytotoxicity comparable to that of the nonfunctionalized system (29% viability; ∼2.3‐fold LDH), indicating that targeting did not alter therapeutic potency. EGFR blocking nearly abolished the efficacy of the GE11‐functionalized formulation, increasing viability to ∼81% and reducing LDH release to ∼1.2‐fold, confirming that GE11‐EGFR interactions mediated internalization and gene delivery.

The relatively high cancer cell uptake observed even with the nonfunctionalized GO formulation is consistent with our previous 2D monoculture observations [37]. A549 cells exhibit elevated nanocarrier internalization, attributable to high basal endocytic activity and increased expression of surface receptors involved in nanoparticle uptake, rendering them particularly permissive to GO internalization [76, 77]. THP1‐derived macrophages typically exhibit high nanoparticle uptake via phagocytosis; however, in this system, the small GO flake size (< 200 nm) combined with PEGylation likely reduced immune recognition and opsonization, thereby limiting macrophage uptake despite their phagocytic potential [36, 67, 78, 79].

Fibroblasts in a 2D study exhibited lower GO uptake, likely reflecting reduced basal endocytic activity and ECM barriers that hinder nanoparticle internalization [35]. CAFs have been shown to internalize substantially more nanoparticles than normal fibroblasts. Studies on gold nanoparticles reported up to 10‐fold higher uptake in CAFs, approaching levels observed in cancer cells [80]. Similarly, pancreatic cancer models exhibited ∼50% higher uptake in CAFs with prolonged intracellular retention [81]. These results indicate that CAFs can internalize nanoparticles efficiently in tumor‐like environments. In the AFT model, the lower abundance of fibroblasts (approximately 10‐fold fewer than cancer cells) likely accounts for the detectable yet reduced non‐cancer‐cell uptake. Together, these factors explain why nontargeted GO‐CD47‐ANT1 already exhibits partial cancer cell selectivity, allowing residual uptake by nonmalignant cells internalization. Importantly, this underscores the value of incorporating a targeting ligand to restrict delivery of phagocytosis‐ and apoptosis‐inducing genes to cancer cells, while preserving the nonselective GO–TGFβ component for broad microenvironmental modulation.

Although AFT viability and LDH responses were similar for functionalized and nonfunctionalized formulations under nonblocked conditions, uptake and blocking experiments revealed distinct cellular targets underlying the observed cytotoxicity. This distinction highlights the importance of cell‐type‐resolved analysis in complex 3D tumor models, where comparable bulk efficacy can mask fundamentally different mechanisms of action. Reducing off‐target delivery to stromal and immune compartments is particularly relevant in such systems, as unintended gene modulation in these populations may alter microenvironmental behavior and confound therapeutic interpretation. Thus, GE11 functionalization does not generate cytotoxic efficacy de novo but refines therapeutic selectivity by restricting gene delivery to malignant cells, an effect that may be especially advantageous in vivo, where minimizing unintended immunomodulation is critical for translational safety and efficacy.

#### 3.3.2. GE11 Functionalization Enhanced Cancer Cell Apoptosis

**FIGURE 6:**
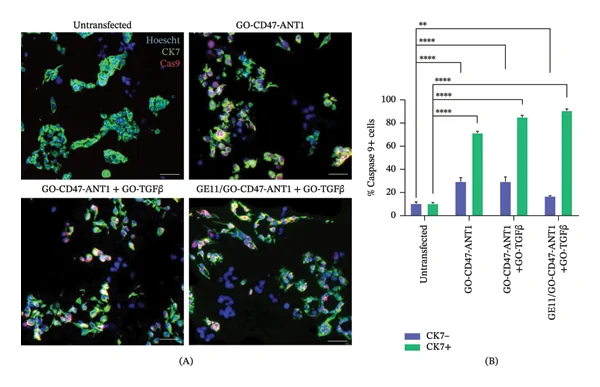
GE11‐mediated targeting enhances cancer‐cell‐selective apoptosis in the AFT model. (A) Representative IHC images of AFT sections (10 μm thick) showing CK7 (green) to identify cancer cells and Caspase 9 (red) as a marker of apoptosis following treatment with GO‐CD47‐ANT1, GO‐CD47‐ANT1_GO‐TGFβ, and GE11/GO‐CD47‐ANT1_GO‐TGFβ, compared with untransfected controls. Nuclei are identified by Hoechst staining (blue). Scale bars: 50 μm. (B) Apoptotic cells quantified as the percentage of CK7^+^ (cancer) or CK7^−^ (noncancer) cells relative to the total number of cells within each population. Statistical significance was assessed using two‐way ANOVA with comparisons made relative to the corresponding cell populations in the untransfected control. ^∗∗^ *p* < 0.01 and ^∗∗∗∗^ *p* < 0.0001 were considered significant, and *p* > 0.05 was considered not significant (not shown).

The impact of GE11‐mediated targeting on apoptotic selectivity was evaluated in the AFT model by comparing apoptosis in cancer versus nonmalignant cells. Figure 6A presents representative IHC sections of untransfected AFT constructs and those transfected with GO‐CD47‐ANT1, GO‐CD47‐ANT1_GO‐TGFβ, and GE11/GO‐CD47‐ANT1_GO‐TGFβ. CK7 staining (green) highlighted cancer cells, while Caspase 9 (red) marked apoptotic cells. Quantitative image analysis (Figure 6B) assessed apoptosis in the CK7^+^ and CK7^−^ populations, enabling evaluation of therapeutic selectivity toward cancer cells.

In untransfected constructs, apoptotic cells were evenly distributed between the CK7^+^ and CK7^−^ populations (10% each), reflecting low background apoptosis and confirming the model’s baseline viability. GO‐CD47‐ANT1 markedly increased apoptosis, with 71% of CK7^+^ cells and 29% of CK7^−^ cells affected, indicating preferential elimination of cancer cells and measurable off‐target effects. The addition of GO‐TGFβ further increased cancer cell apoptosis to 85%, while apoptosis in CK7^−^ cells remained at 29%, consistent with enhanced tumor‐cell susceptibility via immune and stromal modulation.

Notably, after transfection with the GE11/GO‐CD47‐ANT1_GO‐TGFβ formulation, the highest degree of cancer‐cell‐selective apoptosis was observed, with 91% of apoptotic CK7^+^ cells and only 16% of total CK7^−^ cells undergoing apoptosis (Figure 6B). This shift demonstrates that GE11‐mediated targeting substantially reduces apoptosis in nonmalignant cells while enriching apoptotic responses in cancer cells. Importantly, these findings indicate that GE11 functionalization refines cell‐type specificity rather than increasing overall cytotoxic potency. This effect is particularly relevant in multicellular tumor models where off‐target gene delivery may perturb stromal or immune function. In addition to receptor‐mediated targeting, intracellular release mechanisms likely contribute to this selectivity. Previous studies on PEG‐GO and PAMAM‐based systems have shown that mildly acidic environments (pH 5–6), such as those found in endosomal/lysosomal compartments, can promote increased protonation of PAMAM, weakening nucleic acid binding and facilitating intracellular release and endosomal escape via the proton sponge effect. This behavior supports stable complexation at physiological pH while enabling enhanced cargo release under acidic conditions, favoring gene delivery in cancer cells, which often exhibit increased endocytic activity and localized acidic microenvironments [40, 41]. These findings are consistent with prior studies showing that GO‐based nanoplatforms can improve therapeutic efficacy through polymer functionalization, enhanced cancer cell specificity via preferential uptake, and stimulus‐responsive release [35, 67, 73, 77]. For example, polyvinyl alcohol–functionalized magnetic GO has been shown to provide pH‐responsive release of 5‐fluorouracil under tumor‐like acidic conditions, improving targeted drug delivery and cytotoxicity toward breast cancer cells [72]. Similarly, polyvinylpyrrolidone‐functionalized GO enabled efficient dual‐drug loading with pH‐responsive release of quercetin and curcumin, resulting in enhanced anticancer activity compared with single‐drug formulations [73]. Moreover, folate‐functionalized reduced GO loaded with quercetin showed higher drug release under acidic conditions (35% at pH 5.5 vs 20% at pH 7.4 over 48 h), which further increased upon NIR irradiation (up to 70%), resulting in efficient killing of triple‐negative breast cancer cells while sparing normal cells [36]. Similarly, targeted GO platforms with NIR stimulation reduced cancer cell viability to below 20% while sparing normal fibroblasts, demonstrating improved specificity relative to nontargeted systems [82].

Beyond physicochemical targeting, immune‐mediated mechanisms further contribute to cancer cell specificity. In a previous work from our group, in AML co‐cultured with human macrophages, immune activation led to downregulation of CD47 and upregulation of pro‐phagocytic signals on leukemia cells, resulting in selective elimination of malignant cells while sparing normal hematopoietic populations [31].

Collectively, the selectivity observed in the current study reflects the convergence of multiple mechanisms: receptor‐mediated uptake via GE11; a macrophage‐associated therapeutic response linked to CD47 downregulation; apoptosis following delivery of the combined CD47/ANT1 formulation; and microenvironmental sensitization through TGFβ silencing. In the AFT model, these complementary mechanisms act in concert to concentrate apoptotic responses within the malignant compartment while minimizing unintended cytotoxicity in nonmalignant cells, providing a mechanistic rationale for combining targeted nanocarrier delivery with microenvironmental reprogramming in complex tumor systems.

## 4. Conclusions

We report the development and validation of a GO‐based dual gene‐delivery platform designed to address two major barriers in cancer therapy: limited cancer cell specificity and an immunosuppressive, tumor‐supportive microenvironment. Using a multicellular 3D lung cancer model incorporating cancer cells, fibroblasts, and macrophages, we demonstrate that TGFβ silencing alone reprograms stromal and immune components, reducing CAF activation and partially shifting macrophages toward a pro‐inflammatory state, without directly inducing cytotoxicity in cancer cells. In contrast, modulation of CD47 and ANT1 was associated with enhanced cancer cell elimination in macrophage‐containing models, supporting a contribution from macrophages to the therapeutic outcomes. However, phagocytic clearance was not directly quantified in the present study. Combining CD47/ANT1 modulation with TGFβ silencing produced a stronger therapeutic response characterized by increased expression of M1‐associated inflammatory markers, suppression of immunosuppressive cues, and markedly reduced tumor viability. Future studies should incorporate dedicated phagocytosis assays to directly confirm the contribution of macrophage‐mediated clearance to the enhanced therapeutic efficacy observed following CD47 modulation.

Further refinement of the platform through GE11 peptide functionalization of the GO‐CD47‐ANT1 nanocarrier enabled EGFR‐mediated targeting of lung cancer cells, significantly reducing off‐target uptake and apoptosis in nonmalignant populations. Although overall cytotoxicity remained comparable between targeted and nontargeted formulations, cell‐type‐resolved analyses revealed a pronounced shift toward selective apoptosis of cancer cells, underscoring the added value of ligand‐directed delivery in complex multicellular systems.

Collectively, this work establishes a gene‐delivery strategy that integrates direct targeting of malignant cells with concurrent microenvironmental modulation, demonstrating that effective cancer elimination in complex 3D models requires coordinated intervention at both tumor cell and stromal‐immune levels. By leveraging the physicochemical versatility of GO and the complementary mechanisms of macrophage re‐education and CAF suppression, this platform provides a mechanistically grounded and precision‐oriented framework for optimizing cancer‐specific gene therapies in physiologically relevant 3D tumor models.

Despite these promising results, several directions remain to be explored. Although ANT1 transcript upregulation was confirmed by RT‐qPCR, ANT1 protein abundance and downstream mitochondrial activity were not directly evaluated. Since CD47_siRNA and ANT1_pDNA were administered as a combined formulation, the individual contribution of ANT1 to the observed apoptotic and cytotoxic responses cannot be resolved in the present study. Accordingly, these findings are consistent with a contribution of ANT1 to the apoptotic response but do not constitute direct protein level or mitochondrial functional confirmation. Future studies should assess ANT1 protein expression together with mitochondrial endpoints such as membrane potential, respiration, or ATP‐linked bioenergetic changes. Future investigations could further evaluate the impact of this platform on tumor cell migration, invasion, and metastatic potential. Such studies, particularly in larger and more complex tumor models or in vivo systems, will help determine whether the observed stromal and immune reprogramming translates into durable suppression of tumor progression beyond primary tumor elimination. As a nonperfused microscale construct, the AFT model provides a physiologically relevant platform that incorporates cancer, stromal, and immune components within a 3D ECM, yet cannot fully reproduce the transport barriers, vascular heterogeneity, and systemic factors that influence therapeutic performance in vivo, including biodistribution, circulation, immune‐cell trafficking, metabolism, and nanocarrier clearance. The long‐term objective of advanced multicellular tumor models is to improve the predictive value of preclinical testing and reduce reliance on animal experimentation. Nonetheless, at the current stage, larger tissue constructs and in vivo studies remain necessary to establish the translational relevance of these platforms and to evaluate biological processes that cannot be fully captured in vitro. Consequently, future in vivo investigations will be required to validate the therapeutic efficacy, cancer selectivity, and safety profile of the combined GO‐CD47‐ANT1 and GO‐TGFβ strategy, while also providing insight into how accurately the AFT model predicts treatment responses in a physiological setting.

**Nomenclature**
A549: Human lung adenocarcinoma cells
AFT: A549‐fibroblast‐THP1 model
ANT1: Adenine nucleotide translocase 1
ATP: Adenosine triphosphate
CAF: Cancer‐associated fibroblast
CD47: Cluster of differentiation 47
CD68: Cluster of differentiation 68
CD163: Cluster of differentiation 163
CK7: Cytokeratin 7
DC: Dendritic cell
ECM: Extracellular matrix
EGFR: Epidermal growth factor receptor
EMT: Epithelial–mesenchymal transition
GO: Graphene oxide
IHC: Immunohistochemistry
IL1β: Interleukin‐1beta
IL6: Interleukin‐6
IL10: Interleukin‐10
LDH: Lactate dehydrogenase
pDNA: Plasmid DNA
mRNA: RNA messenger
PAMAM: Poly(amidoamine)
PEG: Polyethylene glycol
PMA: Phorbol 12‐myristate 13‐acetate
RT‐qPCR: Reverse transcription quantitative polymerase chain reaction
siRNA: Small interfering RNA
SIRPα: Signal regulatory protein alpha
TAM: Tumor‐associated macrophage
TGFβ: Transforming growth factor‐beta
THP1: human monocytic leukemia cells
TNFα: Tumor necrosis factor‐alpha
αSMA: alpha‐smooth muscle actin

## Author Contributions

Conceptualization: Shan Zou and Francesca Grilli; methodology: Francesca Grilli, Sadman Sakib, and Shan Zou; formal analysis: Francesca Grilli and Sadman Sakib; investigation: Francesca Grilli, Sadman Sakib, and Shan Zou; data curation: Francesca Grilli and Shan Zou; writing−original draft preparation: Francesca Grilli, Sadman Sakib, and Shan Zou; writing−review and editing: All; supervision: Shan Zou and Fabio Variola; project administration: Shan Zou; funding acquisition: Shan Zou.

## Funding

Shan Zou would like to thank the Natural Sciences and Engineering Research Council of Canada through the Discovery Grant (RGPIN‐2021‐03835) for facilitating Francesca Grilli’s stipend.

## Disclosure

All authors have read and agreed to the published version of the manuscript.

## Conflicts of Interest

The authors declare no conflicts of interest.

## Supporting Information

Additional supporting information can be found online in the Supporting Information section.

## Supporting information


**Supporting Information** Quantification analyses of nanocarrier uptake, cell types in AFT models, and macrophage polarization‐associated gene expression levels were included, together with gene expression changes in DC‐added AFT models.

## Data Availability

The data that support the findings of this study are available from the corresponding author upon reasonable request.
