# Supplementary material for: Dual Graphene Oxide–Based Multigene Delivery for Cancer Elimination via Stromal and Immune Reprogramming
Source: Int J Biomater. 2026 Sep 6;2026:3462859. doi: 10.1155/ijbm/3462859 (PMC13547957; doi:10.1155/ijbm/3462859)
Supplement: Supplementary file 1 [file IJBM-2026-3462859-s001.docx]

Supporting Information

Dual Graphene Oxide-Based Multi-Gene Delivery for Cancer Elimination via Stromal and Immune Reprogramming

Francesca Grilli^a,b^, Sadman Sakib^a^, Fabio Variola^b^, and Shan Zou^a,c,*^

^a^ Metrology Research Centre, National Research Council of Canada, 100 Sussex Drive, Ottawa, ON K1A 0R6, Canada.

^b^ Department of Mechanical Engineering, University of Ottawa, 161 Louis Pasteur, Ottawa, ON K1N 6N5, Canada.

^c^ Department of Chemistry, Carleton University, 1125 Colonel By Drive, Ottawa, ON K1S 5B6, Canada.

**GO Nanocarrier Characterization**

The GO nanocarriers used in this study (GO-PEG-PAMAM) have been previously synthesized and extensively characterized in our earlier works [1-6]. Detailed protocols and physicochemical analyses, including AFM for morphology and lateral size, DLS for hydrodynamic size distribution and zeta potential, as well as FTIR, Raman spectroscopy, and XPS for surface chemistry and functionalization, have been reported. These studies confirm nanoscale dimensions, successful PEG/PAMAM modification, stable physicochemical properties, and gene loading capacity of the GO platform used in the present work.

**Figure S1. Dynamic light scattering (DLS) intensity-weighted size distribution**. GO-PEG-PAMAM (labeled as GO) and GO-PEG-PAMAM/GE11 (GO-GE11 conjugate) nanocarriers dispersed in Milli-Q water (5 μg/mL).

**Table S1.** Zeta potential of different formulations

| Formulations | GO | GE11 | GO + GE11 (mix) | GO-GE11 (conjugated) | Comment |
| --- | --- | --- | --- | --- | --- |
| Zeta potential (mV) | +18 | -2 | 24 | -1 | GO was modified with PEG and PAMAM |
| SD (n=5) (mV) | 3 | 3 | 6 | 4 |  |

In this study, additional characterization (Figure S1 and Table S1) was performed to verify GE11 peptide functionalization. Zeta potential measurements (Table S1) were carried out on GO (GO-PEG-PAMAM: ~ +18 mV), GE11 (~ −5 to +1 mV), physical mixtures of GO+GE11 and functionalized GO-GE11 nanocarriers. Simple mixtures of GO and GE11 at different ratios showed increasingly positive values (~+18 to +30 mV). In contrast, GO-GE11 nanocarriers exhibited a clear shift toward near-neutral or slightly negative zeta potentials (~+3 to −5 mV), consistent with surface modification and particle charge neutralization following peptide conjugation. These results confirm the successful functionalization of GO with GE11.

Zeta potential is an operationally defined measurand reflecting the potential at the shear plane rather than the true surface charge. Its magnitude depends on the electrical double layer and measurement conditions (e.g., ionic strength, electrolyte composition, pH, and applied field). Therefore, values should be interpreted with caution and compared only under consistent conditions.

**SYBR Green qPCR melt curves**

**Figure S2. Representative SYBR Green qPCR melt curves for target and reference genes used in this study.** Single melting peaks were observed for all primer sets, confirming amplification specificity and the absence of significant non-specific products or primer-dimer formation. Representative melt curves are shown from control samples.

**Cells quantification**

**Figure S3. Cell type quantification in the AFT model.** A) IHC images of 10 μm thick sections from the central region of the AFT model. Blue: Hoechst nuclei; green: CK7^+^ cancer cells; red: CD68^+^ THP1-derived macrophages. Fibroblasts are identified as cells double negative to CK7 and CD68 staining (CK7^-^/CD68^-^, white arrows). Scale bars = 170 μm. B) Total cells quantified as the percentage of CK7⁺ (cancer), CD68^+^ (THP1-derived macrophages) and CK7⁻/CD68^-^ (fibroblasts) cells relative to the total number of cells. Quantification was performed by analyzing three sections of three models from three independent biological replicates (n=3).

**Macrophages polarization-associated gene expression (M0, M1, M2)**

**Figure S4. TGFβ involvement in macrophage activation.** qPCR of macrophage polarization cytokines and markers after PMA treatment and 72 h transfection with GO-TGFβ, GO-CD47-ANT1 and GO-CD47-ANT1 + GO-TGFβ. Heatmap displays M0 (CD68), M1 (IL1β, TNFα, IL6) and M2 (CD163, IL10, TGFβ) gene expression. White arrows represent the upregulation or downregulation of mRNA levels related to untransfected control. Statistical significance was determined against the untransfected AFT model using two-way ANOVA (*P<0.05; **P<0.01; ***P<0.001; and ****P<0.0001; ns: P>0.05, not shown).

**Gene expression regulation in DCs containing models**

**Table S2.** Fold changes in CD47, ANT1 and TGFβ gene expression in AFDC and AFTDC models following transfection with GO-CD47-ANT1 + GO-TGFβ. Values are reported as mean ± SD. Statistical significance was assessed relative to the respective untransfected control, using two-way ANOVA, with **P< 0.01 considered significant. P> 0.05 was considered not significant.

| Gene expression fold change | CD47 | ANT1 | TGFβ |
| --- | --- | --- | --- |
| AF-DC | 0.4 ± 0.1 (**) | 2.1 ± 0.2 | 0.3 ± 0.1 (**) |
| AFT-DC | 0.7 ± 0.1 (**) | 2 ± 0.3 (**) | 0.5 ± 0.1 (**) |

**GO-based nanocarriers uptake**

**Table S3.** CD47-ANT1 nanocarrier component uptake by CK7^+^ cancer cells and CK7^-^ non-cancer cells in the AFT model, following treatment with GO-CD47-ANT1 + GO-TGFβ and **GE11**/GO-CD47-ANT1 + GO-TGFβ in the absence or presence of EGFR blocking through incubation with extra GE11 free peptide. Values are reported as mean ± SD.

| %Uptake | CK7^+^ | CK7^-^ |
| --- | --- | --- |
| GO-CD47-ANT1 + GO-TGFβ | 82 ± 1 | 35 ± 2 |
| GO-CD47-ANT1 + GO-TGFβ  + GE11 blocking | 80 ± 2 | 36 ± 2 |
| GE11/GO-CD47-ANT1 + GO-TGFβ | 90 ± 1 | 14 ± 1 |
| GE11/GO-CD47-ANT1 + GO-TGFβ + GE11 blocking | 23 ± 2 | 14 ± 1 |

**References**

[1] Coleman, B. R.; Knight, T.; Gies, V.; Jakubek, Z. J.; Zou, S. Manipulation and Quantification of Graphene Oxide Flake Size: Photoluminescence and Cytotoxicity. *ACS Appl Mater Interfaces* **2017**, *9* (34), 28911-28921. DOI: 10.1021/acsami.7b08585

[2] Gies, V.; Zou, S. Systematic toxicity investigation of graphene oxide: evaluation of assay selection, cell type, exposure period and flake size. *Toxicol Res* **2018**, *7* (1), 93-101. DOI: 10.1039/c7tx00278e

[3] Gies, V.; Lopinski, G.; Augustine, J.; Cheung, T.; Kodra, O.; Zou, S. The impact of processing on the cytotoxicity of graphene oxide. *Nanoscale Adv* **2019**, *1* (2), 817-826. DOI: 10.1039/c8na00178b

[4] Hassan, E. M.; Zou, S. Novel nanocarriers for silencing anti-phagocytosis CD47 marker in acute myeloid leukemia cells. *Colloid Surface B* **2022**, *217*, 112609. DOI: 10.1016/j.colsurfb.2022.112609

[5] Hassan, E. M.; McWhirter, S.; Walker, G. C.; Martinez-Rubi, Y.; Zou, S. Elimination of Cancer Cells in Co-Culture: Role of Different Nanocarriers in Regulation of CD47 and Calreticulin-Induced Phagocytosis. *ACS Appl Mater Interfaces* **2023**, *15* (3), 3791-3803. DOI: 10.1021/acsami.2c19311

[6] Sakib, S.; Zou, S. Attenuation of Chronic Inflammation in Intestinal Organoids with Graphene Oxide-Mediated Tumor Necrosis Factor-alpha_Small Interfering RNA Delivery. *Langmuir* **2024**, *40* (7), 3402-3413. DOI: 10.1021/acs.langmuir.3c02741
